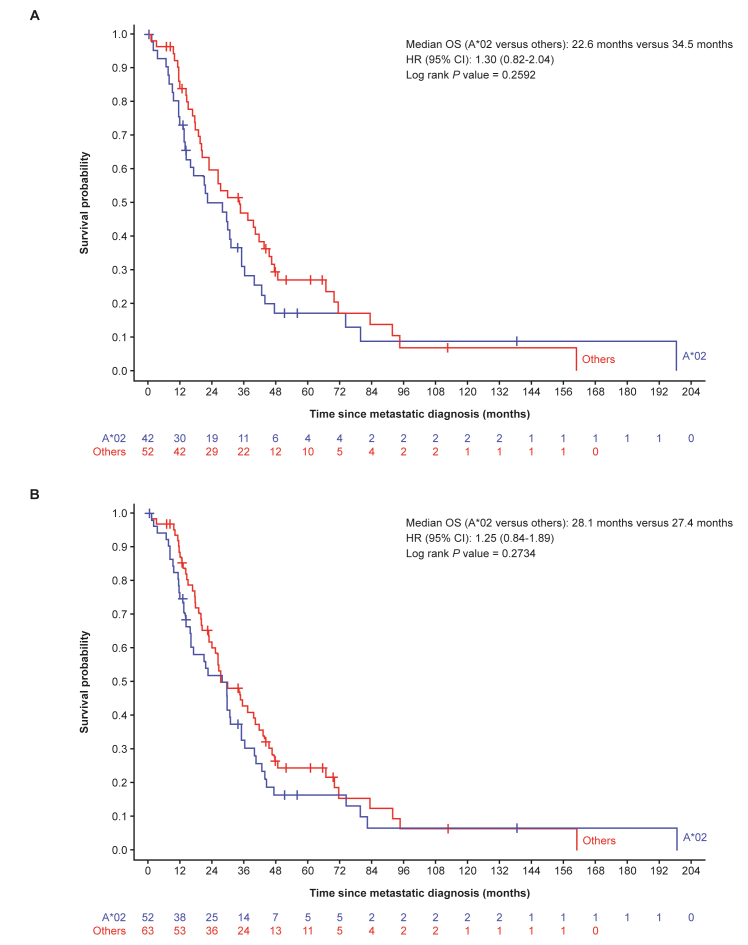# Investigating the prognostic impact of NY-ESO-1 expression and HLA subtypes in metastatic synovial sarcoma

**DOI:** 10.1016/j.esmoop.2024.103645

**Published:** 2024-08-16

**Authors:** A. Dufresne, S. Pokras, A. Meurgey, S. Chabaud, M. Toulmonde, E. Bompas, A. Le Cesne, Y.-M. Robin, F. Duffaud, T. Valentin, S. El Zein, A. Leroux, P. Dubray-Longeras, N. Firmin, G. de Pinieux, S. Noal, C. Delfour, J. Bollard, L. Tonon, A. Biette, N. Gadot, V. Attignon, M. Jean-Denis, M. Woessner, E. Klohe, T. Thayaparan, I. Eleftheriadou, K. Blouch, M.J. Nathenson, J.-Y. Blay

**Affiliations:** 1 Department of Medical Oncology, Centre Léon Bérard, Lyon, France; 2 GSK, Collegeville, USA; 3 Department of Pathology, Centre Léon Bérard, Lyon; 4 Department of Clinical Research and Innovation, Centre Léon Bérard, Lyon; 5 Department of Medical Oncology, Institut Bergonié, Bordeaux; 6 Department of Medicine, Institut Cancérologie de l’Ouest, Maine Et Loire; 7 Department of Medicine, Gustave Roussy, Villejuif; 8 Department of Biopathology, Centre Oscar Lambret, Lille; 9 Department of Medical Oncology, Assistance Publique des Hôpitaux de Marseille, Hôpital La Timone, Aix-Marseille University, Marseille; 10 Medical Oncology, Institut Claudius Regaud, Institut Universitaire du Cancer de Toulouse, Toulouse; 11 Department of Pathology, Institut Curie and Paris Sciences Lettres University, Île-de-France; 12 Department of Biopathology, Institut de Cancérologie de Lorraine, Vandœuvre-lès-Nancy; 13 Department of Medical Oncology, Centre Jean Perrin, Clermont-Ferrand; 14 Department of Medical Oncology, Institut du Cancer de Montpellier, Institut de Recherche en Cancérologie de Montpellier U1194, Université de Montpellier, Montpellier; 15 Department of Pathology, Université de Tours, Tours; 16 Centre François Baclesse, Caen; 17 Department for Cell and Tissue Pathobiology of Tumor, Hospital Saint Eloi, Montpellier; 18 Department of Research and Innovation, Centre Léon Bérard, Lyon; 19 Plateforme de Bioinformatique Gilles Thomas, Fondation Synergie Lyon Cancer, Centre Léon Bérard, Lyon; 20 Evaluation Médicale des Sarcomes, Centre Léon Bérard, Lyon; 21 Research Pathology Platform, Centre Léon Bérard, Lyon, France

**Keywords:** metastatic synovial sarcoma, NY-ESO-1 expression, HLA subtypes, adoptive T-cell therapy

## Abstract

**Background:**

To better understand the importance of New York esophageal squamous cell carcinoma 1 (NY-ESO-1) and human leukocyte antigen (HLA) subtype in treatment decision making, further investigation of their prevalence and prognostic impact among patients with metastatic synovial sarcoma (mSS) is needed.

**Patients and methods:**

This was a retrospective clinico-biological cohort study of adults with mSS. Patient data were collected from the French Sarcoma Group NetSARC database and supplemented by electronic medical records. Primary tumor samples were collected and analyzed for NY-ESO-1 expression by immunohistochemistry (IHC) and HLA-A∗02 status by RNA sequencing (RNA-seq). The primary cohort included patients with available primary tumor samples; the impact of a larger sample size was explored by including patients who had either a primary or metastatic sample (termed the exploratory cohort). *P* values are provided for descriptive purposes.

**Results:**

In 92 patients with primary tumor samples, ∼25% (*n* = 23) were positive for NY-ESO-1 and HLA-A∗02 expression (dual positive). Among 106 patients with IHC data, 61% (*n* = 65) were NY-ESO-1 positive, and among 94 patients with RNA-seq data, 45% (*n* = 42) were HLA-A∗02 positive. The median overall survival (OS) for positive versus negative NY-ESO-1 status was 35.3 and 21.7 months, respectively (unadjusted *P* = 0.0428). We observed no difference in median OS for HLA-A∗02-positive versus -negative and dual-positive patients versus others (both unadjusted *P* > 0.05). Multivariate analyses of OS showed no prognostic impact for NY-ESO-1 among primary tumor samples and in the exploratory cohort. However, in the latter, we observed an association between NY-ESO-1 expression and OS in the first-line (*P* = 0.0041) but not in the second-line setting.

**Conclusions:**

The primary tumor cohort showed no association between NY-ESO-1 expression and OS (including stratification by HLA-A∗02 subtype and treatment line), when adjusting for important prognostic factors, possibly due to small sample sizes.

## Highlights

• This is the first study to examine the prevalence and prognostic impact of NY-ESO-1 and HLA-A subtypes in patients with mSS.
• Sixty-one percent and 45% of patients were positive for NY-ESO-1 and HLA-A subtype, respectively; 25% were dual positive.
• There was no prognostic impact of NY-ESO-1 expression for OS, except in the exploratory cohort first-line setting.
• There was no difference in OS by HLA-A subtype and when combined with NY-ESO-1 expression.
• OS in patients with mSS was low, irrespective of NY-ESO-1 or HLA-A expression or treatment line.

## Introduction

Synovial sarcomas (SSs) comprise ∼5%-10% of all soft tissue sarcomas (STSs).^1^ SS is characterized by translocation t(X;18)(p11.2;q11.2), which results in the fusion of genes *SSX1*, *SSX2*, or *SSX4*, and *SYT* (*SS18*).^2^^,^^3^ SS usually presents in the lower extremities of adolescents and young adults.^1^ Although most patients are diagnosed with localized disease, around 50% of them will develop metastatic disease.^4^^,^^5^

Chemotherapy is the standard treatment in metastatic or unresectable disease and typically comprises anthracycline and/or ifosfamide as first line (L1); however, this approach has been shown to have limited efficacy.^1^ Treatment options in the second-line (L2) setting for advanced SS include trabectedin and the oral angiogenesis inhibitor pazopanib.^6^ Pazopanib has been reported to provide a progression-free survival (PFS) rate of 49% at 12 weeks in patients with advanced SS; nevertheless, no complete responses have been observed.^7^ The need for novel treatments with improved efficacy was highlighted by a recent meta-analysis of 16 studies with pazopanib or trabectedin in previously treated patients with metastatic SS (mSS), in which the overall estimate of pooled objective response rate was 14.7% and median overall survival (OS) was <12 months for both medications.^8^

New York esophageal squamous cell carcinoma 1 (NY-ESO-1) cancer testis antigens are tumor-associated proteins that have been found specifically expressed in several tumor types, including a sizable proportion of SS.^9^^,^^10^ NY-ESO-1 has been investigated as a therapeutic target of adoptive cell therapy in several clinical trials, and T-cell receptor gene therapies have been exploring specific human leukocyte antigen (HLA) alleles for presentation of the NY-ESO-1 antigen. However, the prognostic impact of NY-ESO-1 and HLA remains unclear.^11^, ^12^, ^13^, ^14^, ^15^

The literature shows variation in the relationship between NY-ESO-1 expression and OS in STS. One study associated NY-ESO-1 expression with favorable survival prognosis,^11^ one study reported worse survival prognosis,^13^ and three studies have shown that OS is not affected by NY-ESO-1 expression.^12^^,^^15^^,^^16^ However, in these studies, interpretation of the impact of NY-ESO-1 expression on OS is limited by small sample sizes^12^^,^^15^^,^^16^ and/or the absence of SS-specific analyses.^11^^,^^13^

There remains a need for a better understanding of the prevalence of HLA subtype A∗02 (HLA-A∗02) and NY-ESO-1 expression among a broad population of patients with mSS, as well as the prognostic impact of these markers for this patient population. The objectives of this study were to (i) describe the prevalence and levels of expression of NY-ESO-1 and HLA-A∗02 subtypes, and clinical characteristics of adult patients with mSS; and to (ii) describe and compare clinical outcomes among patients with mSS who express NY-ESO-1 and relevant HLA-A∗02 subtypes against those who do not (independently, and together), and by lines of systemic treatment.

## Patients and methods

### Study design

This was a retrospective clinico-biological cohort study in which data were obtained from the NetSARC database of the French Sarcoma Group. The study included adult patients with a histologically confirmed diagnosis of mSS diagnosed since 2000 and with available formalin-fixed paraffin-embedded archival primary tumor samples (and metastatic sample if available) with a corresponding pathological report. The patients were diagnosed and managed in 1 of the 27 national reference centers of the NetSARC network, across France. None of the patients were previously treated with T-cell therapy.

Patient information and tumor characteristics were collected from the NetSARC database, and data from any missing fields and updates were collected from patient electronic medical records from visiting treatment centers. Further information on patient data protection can be found in the Supplementary Methods, available at https://doi.org/10.1016/j.esmoop.2024.103645.

### Biomarker detection

Patients with mSS were identified from the French Sarcoma Group database and each case was reviewed by a sarcoma expert pathologist. NY-ESO-1 antigen expression on primary tumor sample was assessed using immunohistochemistry (IHC). Determination of NY-ESO-1 positivity was based on the diagnostic enrolment criteria of ≥30% tumor cell staining at 2+/3+ intensity. HLA-A∗02 positivity was determined by RNA sequencing of the primary tumor sample and included patients with HLA-A∗02:01, HLA-A∗02:05, or HLA-A∗02:06 status. Further information on tissue collection, validation, and concordance assessment of the Centre Léon Bérard NY-ESO-1 IHC assay can be found in the Supplementary Methods, available at https://doi.org/10.1016/j.esmoop.2024.103645.

### Study cohorts

While the pre-planned primary analysis was conducted among patients with primary tumor samples available (termed the primary cohort), *ad hoc* exploratory analyses were conducted in patients with either a primary or metastatic sample (termed the exploratory cohort; for patients with both primary and metastatic samples, the metastatic sample was selected) to explore the effect of an increase in sample size on results.

### Endpoints

The study endpoints included OS, PFS, and objective response rate, measured as per best RECIST 1.1 criteria, and time to next treatment among patients with mSS with NY-ESO-1 and HLA-A∗02 subtype positivity against those who do not (independently, and together), and by lines of systemic treatment. This study focuses on OS analyses of patients with mSS according to NY-ESO-1 expression, HLA-A∗02 subtypes, and lines of systemic treatment. Patients who were NY-ESO-1 positive and HLA∗A02 positive were termed ‘dual positive’.

### Statistical analysis

Clinical covariates assessed included age at diagnosis of metastatic disease, sex, histology, size, grade, and location of primary tumor, time between original and metastasis diagnosis, year of metastasis diagnosis, site of first metastasis, number of metastatic sites, number of chemotherapy lines, and drugs used in L1 systemic therapy.

For the ‘overall’ patient population, which included treated and untreated patients, irrespective of the line of systemic treatment, OS was measured from date of metastatic diagnosis to date of death, or date of last news for patients still alive, at the time of database lock (1 August 2021). For patients receiving L1 and L2 systemic therapy, OS was calculated from systemic therapy initiation until date of death, or date of last news for patients still alive, at database lock.

As the final sample size was determined by the availability of tissue and completeness of data, and the a priori hypothesis was that HLA subtype and NY-ESO-1 positivity have no effect on prognosis, a sample size or power calculation would have little benefit. Instead, the calculation of the minimum detectable effect (MDE) was implemented. At a given significance level, this estimate could yield a significant result that could distinguish survival between the different subtypes.

The MDE is a function of total sample size, the proportion of events expected across the sample, the proportion of the sample allocated to the positive subgroup, and the type 1 and type 2 errors. The proportion of events expected across the sample was expected to be 70% across all scenarios. Type 1 and type 2 errors were assumed to be 5% and 20%, respectively. The proportion of sample allocated to the positive subgroup was likely to be 50% for patients who were HLA-A∗02 positive and 70% for patients who were NY-ESO-1 positive. The scenario in which 70% of patients are NY-ESO-1 positive also applies to patients who are HLA-A∗02/NY-ESO-1 positive, which is expected to represent ∼35% of patients. Based on a sample size of 100, the maximum detectable hazard ratio (HR) yielding a significant difference in survival between positive and negative subgroups was 0.63 for HLA-A∗02 and 0.60 for NY-ESO-1.

Statistical analyses of patient demographics and characteristics of patients in the dual-positive cohort versus all metastatic patients were carried out using Fisher’s exact test. Several tests were conducted without adjustments for multiple testing; alpha adjustments and a predefined testing hierarchy were not included.

Survival distributions were estimated using the Kaplan–Meier method and compared between biomarker expression groups using the log-rank test. Univariate analyses were used to evaluate the prognostic value of each covariate and biomarker on OS. Covariates found to be significant at 0.1 alpha level in the univariate analysis were then included in the multivariate model. All multivariate analyses *P* values were calculated by Wald chi-square test.

All analyses were provided with corresponding *P* values for descriptive purposes and were evaluated against a nominal *P* value of 0.05 for exploratory purposes. All statistical analyses were carried out using SAS® software, v9.4 (SAS Institute Inc, Cary, NC).

## Results

### Patient prevalence and characteristics

**Figure 1:**
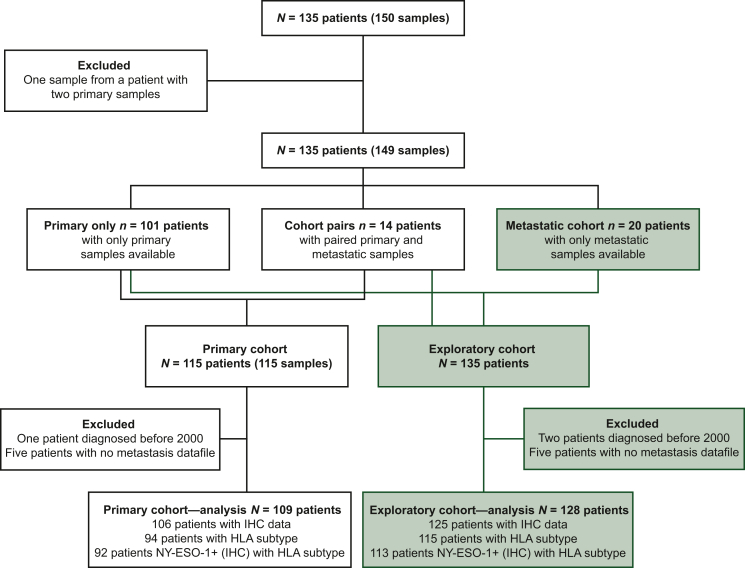
**CONSORT diagram of primary and exploratory patient populations.** CONSORT, Consolidated Standards of Reporting Trials; HLA, human leukocyte antigen; IHC, immunohistochemistry; NY-ESO-1, New York esophageal squamous cell carcinoma 1.

The primary cohort dataset for analysis comprised 109 patients, and 128 patients were included in the exploratory cohort (Figure 1). The prevalence of NY-ESO-1 and HLA-A∗02 positivity is shown in Table 1. The proportion of patients who were NY-ESO-1 positive was 61% (65/106), with similar NY-ESO-1 positivity among the HLA-A∗02-positive and HLA-A∗02-negative populations. In addition, 45% (42/94) of patients were HLA-A∗02 positive, with similar HLA-A∗02 positive prevalence among NY-ESO-1-positive and NY-ESO-1-negative populations. Overall, 25% (23/91) were dual positive.

**Table 1:** Overall prevalence of NY-ESO-1 expression and HLA-A∗02 subtypes by sample size (primary cohort)

	NY-ESO-1+	NY-ESO-1−	Missing data	Total
HLA+	23 (25.3)	18 (19.8)	1	42 (44.7)
HLA−	31 (34.1)	19 (20.9)	2^a^	52 (55.3)
Missing data	11	4	0	15
Total	65 (61.3)	41 (38.7)	3	109

HLA+ refers to HLA-A∗02. All values are *n* (%).

HLA, human leukocyte antigen; HLA-A, human leukocyte antigen-A; IHC, immunohistochemistry; NY-ESO-1, New York esophageal squamous cell carcinoma 1.

a One of the two patients with missing data for HLA had available IHC data but their NY-ESO-1 result was not informative. This patient was included in overall survival analyses of the dual-positive group versus others.

At the time of data analysis, 80.7% (*n* = 88) of patients had died and the median follow-up was 69 months (range 1.1-138 months). Out of 109 patients, 45% (*n* = 49) were female and 45.9% (*n* = 50) were ≤45 years of age at metastatic diagnosis. Table 2 reports the distribution of tumor, patient, and management characteristics in the primary cohort comparing dual-positive patients with others. The proportion of patients with location of the primary tumor in the limbs [*n* = 20 (87%), *P* = 0.003] and location of metastases only in the lungs [*n* = 21 (91.3%), *P* = 0.029] was higher for the dual-positive group versus the total population (Table 2). Nine patients (8.3%) did not receive any chemotherapy, whereas 20 (18.3%) and 80 (73.4%) patients received L1 and L2+ chemotherapy, respectively.

**Table 2:** Clinical characteristics of patients in the dual-positive cohort versus others, among all metastatic patients (primary cohort)

	Missing data	NY-ESO-1+/HLA+	Others	All	Fisher’s exact test
	*N* = 17	*N* = 23	*N* = 69	*N* = 109	
Sex					
Female	11 (64.7%)	9 (39.1%)	29 (42.0%)	49 (45.0%)	*P* = 1.000
Male	6 (35.3%)	14 (60.9%)	40 (58.0%)	60 (55.0%)	
Primary tumor size (mm)					
Missing data	1	3	0	4	
<50	1 (6.3%)	2 (10.0%)	9 (13.0%)	12 (11.4%)	*P* = 1.000
≥50	15 (93.8%)	18 (90.0%)	60 (87.0%)	93 (88.6%)	
Histology					
Synovial sarcoma	2 (11.8%)	3 (13.0%)	9 (13.0%)	14 (12.8%)	*P* = 0.644
Synovial sarcoma—poorly differentiated	2 (11.8%)	1 (4.3%)	8 (11.6%)	11 (10.1%)	
Synovial sarcoma mono- and bi-phasic	13 (76.5%)	19 (82.6%)	52 (75.4%)	84 (77.1%)	
Site of primary tumor					
Members	11 (64.7%)	20 (87.0%)	34 (49.3%)	65 (59.6%)	***P* = 0.003**
Others	2 (11.8%)	0 (0.0%)	16 (23.2%)	18 (16.5%)	
Trunk	4 (23.5%)	3 (13.0%)	19 (27.5%)	26 (23.9%)	
Metastatic-free interval					
Missing data	0	1	0	1	
M0 ≤ 12 months	2 (11.8%)	9 (40.9%)	20 (29.0%)	31 (28.7%)	*P* = 0.316
M0 > 12 months	10 (58.8%)	7 (31.8%)	35 (50.7%)	52 (48.1%)	
M1	5 (29.4%)	6 (27.3%)	14 (20.3%)	25 (23.1%)	
Age at metastatic diagnosis					
≤45 years	9 (52.9%)	12 (52.2%)	29 (42.0%)	50 (45.9%)	*P* = 0.471
>45 years	8 (47.1%)	11 (47.8%)	40 (58.0%)	59 (54.1%)	
Year of metastatic diagnosis					
≤2010	6 (35.3%)	15 (65.2%)	28 (40.6%)	49 (45.0%)	*P* = 0.054
>2010	11 (64.7%)	8 (34.8%)	41 (59.4%)	60 (55.0%)	
Site of first metastasis					
Lung only	9 (52.9%)	21 (91.3%)	46 (66.7%)	76 (69.7%)	***P* = 0.029**
Other	8 (47.1%)	2 (8.7%)	23 (33.3%)	33 (30.3%)	
Number of metastatic sites					
1	14 (82.4%)	22 (95.7%)	59 (85.5%)	95 (87.2%)	*P* = 0.280
2+	3 (17.6%)	1 (4.3%)	10 (14.5%)	14 (12.8%)	
Number of chemotherapy lines					
0	3 (17.6%)	3 (13.0%)	3 (4.3%)	9 (8.3%)	*P* = 0.345
1	5 (29.4%)	3 (13.0%)	12 (17.4%)	20 (18.3%)	
2+	9 (52.9%)	17 (73.9%)	54 (78.3%)	80 (73.4%)	
Clinical trial in L1					
No	13 (76.5%)	19 (82.6%)	53 (76.8%)	85 (78.0%)	*P* = 0.097
No CT line 1	3 (17.6%)	3 (13.0%)	3 (4.3%)	9 (8.3%)	
Yes	1 (5.9%)	1 (4.3%)	13 (18.8%)	15 (13.8%)	
Drug used in L1					
Missing data	0	1	2	3	
All dox + ifos combinations	6 (35.3%)	9 (40.9%)	20 (29.9%)	35 (33.0%)	*P* = 0.570
Dox alone or combination	0 (0.0%)	3 (13.6%)	14 (20.9%)	17 (16.0%)	
Ifos alone or combination	3 (17.6%)	3 (13.6%)	10 (14.9%)	16 (15.1%)	
No CT L1	3 (17.6%)	3 (13.6%)	3 (4.5%)	9 (8.5%)	
Others	1 (5.9%)	3 (13.6%)	10 (14.9%)	14 (13.2%)	
Pazopanib/VEGFs alone or combination	2 (11.8%)	1 (4.5%)	4 (6.0%)	7 (6.6%)	
Trabectedin alone or combination	2 (11.8%)	0 (0.0%)	6 (9.0%)	8 (7.5%)	
Death					
No	4 (23.5%)	7 (30.4%)	10 (14.5%)	21 (19.3%)	*P* = 0.120
Yes	13 (76.5%)	16 (69.6%)	59 (85.5%)	88 (80.7%)	
Progression or death in L1					
Missing data	3	4	5	12	
No	6 (42.9%)	5 (26.3%)	10 (15.6%)	21 (21.6%)	*P* = 0.317
Yes	8 (57.1%)	14 (73.7%)	54 (84.4%)	76 (78.4%)	
Progression or death in L2					
Missing data	10	10	23	43	
No	0 (0.0%)	0 (0.0%)	3 (6.5%)	3 (4.5%)	*P* = 1.000
Yes	7 (100.0%)	13 (100.0%)	43 (93.5%)	63 (95.5%)	

Others include patients who were positive for NY-ESO-1 and negative for HLA subtype A∗02, negative for NY-ESO-1 and positive for HLA subtype A∗02, and negative for NY-ESO-1 and negative for HLA subtype A∗02. HLA+ refers to HLA-A∗02. *P* values below 0.05 are given in bold.

CT, chemotherapy treatment; dox, doxorubicin; HLA, human leukocyte antigen; HLA-A, human leukocyte antigen-A; ifos, ifosfamide; L1, first line; L2, second line; NY-ESO-1, New York esophageal squamous cell carcinoma 1; VEGF, vascular endothelial growth factor.

### Primary and metastatic paired sample summary

Fourteen patients had paired primary and metastatic samples available (Supplementary Table S1, available at https://doi.org/10.1016/j.esmoop.2024.103645). Although there was high concordance in NY-ESO-1 positivity between patients’ metastatic and primary tumor samples (10/14), it was not fully concordant. In three cases (21.4%), NY-ESO-1 expression was positive in the metastatic sample but negative in the primary sample. In one case (7.1%), NY-ESO-1 expression was negative in the metastatic sample but positive in the primary sample. In these four cases, patients received systemic chemotherapy between primary and metastatic sampling. Therefore, both primary and exploratory cohort results are reported.

### Overall survival

#### Overall survival according to NY-ESO-1 positivity

Median OS in the overall primary cohort (*n* = 109) was 26.4 months [95% confidence interval (95% CI) 19.1-35.1 months].

**Figure 2:**
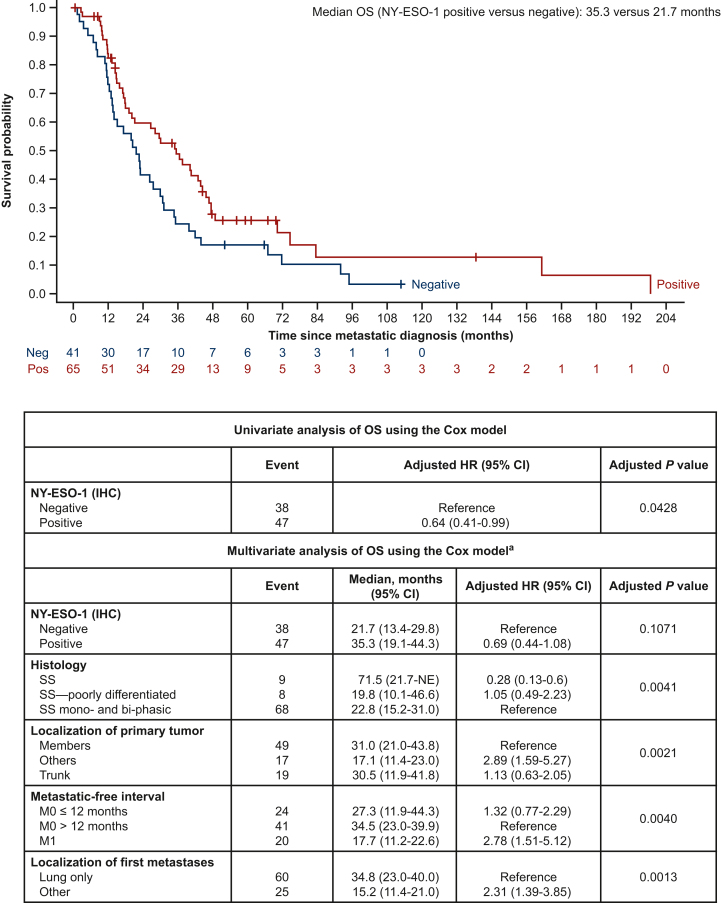
**OS according to NY-ESO-1 positivity by IHC in the primary cohort.** All multivariate analysis *P* values were calculated by Wald chi-square test. CI, confidence interval; HR, hazard ratio; IHC, immunohistochemistry; NE, not evaluable; NY-ESO-1, New York esophageal squamous cell carcinoma 1; OS, overall survival; SS, synovial sarcoma. ^a^Final model is estimated on 105 patients and 85 deaths due to missing values.

Median OS for patients positive for NY-ESO-1 was 35.3 months (95% CI 19.1-44.3 months) versus 21.7 months (95% CI 13.4-29.8 months) for those who were negative (Figure 2). The unadjusted HR for univariate analysis of OS was 0.64 (95% CI 0.41-0.99, unadjusted *P* = 0.0428). Multivariate analysis of OS revealed no significant prognostic value of NY-ESO-1 IHC expression, after adjustment on histology, site of primary tumor, metastatic-free interval, and localization of first metastases [adjusted HR 0.69 (95% CI 0.44-1.08), adjusted *P* = 0.1071]. Analysis by line of treatment also revealed no association upon multivariate adjustment in the primary cohort (Supplementary Tables S2 and S3, available at https://doi.org/10.1016/j.esmoop.2024.103645).

**Supplementary Figure 1:**
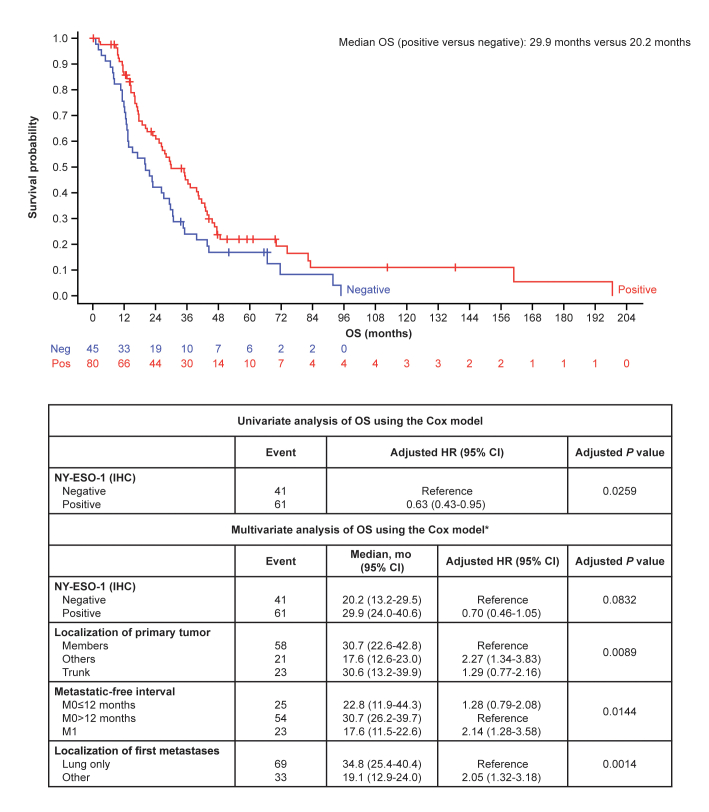


In the exploratory cohort, median OS for patients positive for NY-ESO-1 was 29.9 months (95% CI 24.0-40.6 months) versus 20.2 months (95% CI 13.2-29.5 months) for those who were negative (Supplementary Figure S1, available at https://doi.org/10.1016/j.esmoop.2024.103645). Univariate and multivariate analyses of the median OS for patients positive for NY-ESO-1 were comparable to the primary cohort [unadjusted HR 0.63 (95% CI 0.43-0.95), unadjusted *P* = 0.0259; adjusted HR 0.70 (95% CI 0.46-1.05), adjusted *P* = 0.0832, respectively] (Supplementary Figure S1, available at https://doi.org/10.1016/j.esmoop.2024.103645). However, for the metastatic L1 setting in the exploratory cohort, NY-ESO-1 positivity was associated with improved median OS, despite multivariate adjustment [HR 0.52 (95% CI 0.34-0.82), *P* = 0.0041].

#### Overall survival according to HLA-A∗02 positivity

Median OS for patients positive for HLA-A∗02 was 22.6 months (95% CI 13.9-35.1 months) versus 34.5 months (95% CI 20.2-43.7 months) for others [unadjusted HR 1.30 (95% CI 0.82-2.04), unadjusted *P* = 0.2592] (Supplementary Figure S2A, available at https://doi.org/10.1016/j.esmoop.2024.103645). There was no association observed between HLA-A∗02 positivity and OS, including by line of treatment. Similar results were reported in the exploratory cohort (Supplementary Figure S2B, available at https://doi.org/10.1016/j.esmoop.2024.103645).

#### Overall survival according to dual-positive expression

**Figure 3:**
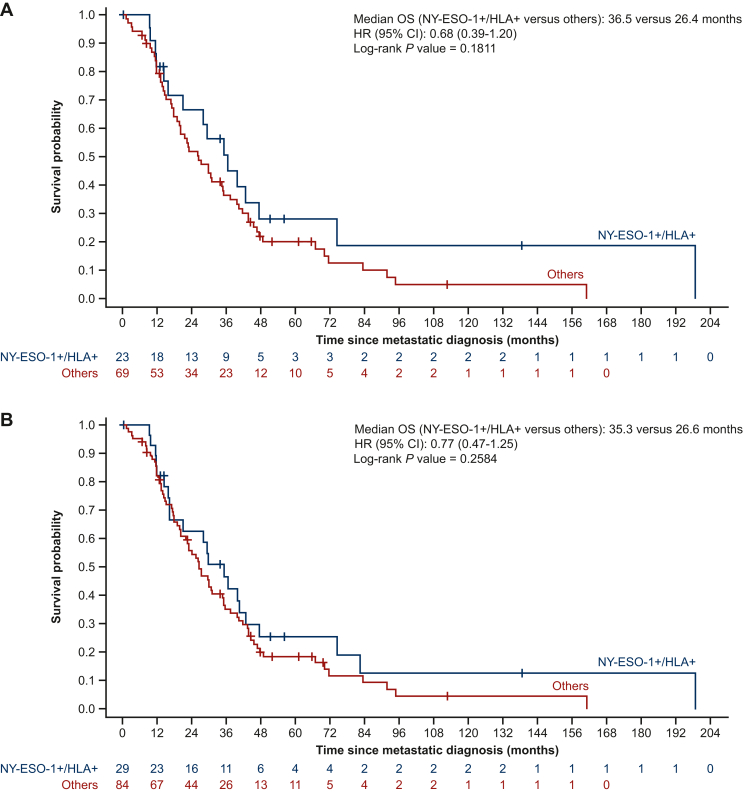
**OS according to dual positivity in the primary (A) and exploratory (B) cohorts.** Data show median OS for patients who were positive for NY-ESO-1 and had HLA-A subtype A∗02 versus those who were negative for NY-ESO-1 and had other HLA-A subtypes (with unadjusted HR and *P* values). CI, confidence interval; HLA, human leukocyte antigen; HLA-A, human leukocyte antigen-A; HR, hazard ratio; IHC, immunohistochemistry; NY-ESO-1, New York esophageal squamous cell carcinoma 1; OS, overall survival.

Median OS for patients with dual-positive expression was 36.5 months (95% CI 15.9-47.4 months) versus 26.4 months (95% CI 19.1-34.8 months) for others [unadjusted HR 0.68 (95% CI 0.39-1.20), unadjusted *P* = 0.1811] (Figure 3A). There was no association observed between dual positivity and OS, including by line of treatment. Similar results were reported in the exploratory cohort, in which the median OS for patients with dual-positive expression was 35.3 months (95% CI 16.2-42.8 months) versus 26.6 months (95% CI 20.2-34.5 months) for others [unadjusted HR 0.77 (95% CI 0.47-1.25), unadjusted *P* = 0.2584] (Figure 3B).

## Discussion

Assessment of the prevalence of NY-ESO-1 expression and HLA-A∗02 subtypes is crucial for the further clinical development of agents targeting these biomarkers in mSS. This is especially relevant with adoptive T-cell therapies, in which assessment of efficacy through randomized trials is challenging. In this context, it is essential to confirm that expression of NY-ESO-1 and HLA-A∗02 subtypes does not modify the natural history of the disease. To our knowledge, this is the largest clinico-biological cohort to examine the prevalence and the prognostic impact of both NY-ESO-1 and HLA-A subtypes in patients with mSS, and the first study in this population using a combination of real-world data and archival tissue samples.

In this study, 61% (*n* = 65) of patients were NY-ESO-1 positive and 45% (*n* = 42) were HLA-A∗02 positive; 25% were dual positive, with comparable prevalence in L1 and L2 settings. Previous IHC studies reported a generally higher prevalence of NY-ESO-1 expression in SS tissue samples (71%-82%) than that observed in our study.^17^, ^18^, ^19^

High concordance in NY-ESO-1 positivity between patients’ primary and metastatic tumor samples was observed, but a lack of total concordance highlighted the importance of including both primary and exploratory cohort results.

The median OS in the primary tumor cohort was short (26.4 months) in the mSS population, irrespective of NY-ESO-1 or HLA-A∗02 positivity or treatment line. Our findings on OS are comparable with recent data from the French Sarcoma Group using the same database in patients with mSS (median OS of 22.3 months).^20^ Unadjusted results reported a longer OS in patients whose tumors expressed NY-ESO-1, although no prognostic impact was observed in the multivariate analysis. In the exploratory cohort, there was an association between NY-ESO-1 positivity and OS since L1 treatment initiation, despite adjusting for certain parameters such as the site of primary tumor, localization of first metastases, and treatment for L1. This may be explained by a subgroup of patients with very good prognostic factors who benefited from surgical approach alone for oligometastatic disease and did not receive any systemic chemotherapy. Therefore, this subgroup was not included in the L1 analysis. There were no differences in survival outcomes based on HLA-A∗02 positivity or on dual positivity, including in the L2 setting.

Several studies have attempted to elucidate the association between NY-ESO-1 expression and OS in STS, with varying conclusions, and each with low sample sizes and/or lack of specificity to an SS population. In a recent study of high-risk patients with STS with long-term follow-up, high NY-ESO-1 expression correlated with favorable survival prognosis (>120 versus 58 months; *P* = 0.0370) and lower tumor grade (grade 2 versus grade 3; *P* = 0.0290); however, this study did not focus on SS specifically.^11^ In a meta-analysis, Wang et al. reported an association between NY-ESO-1 expression and worse OS and PFS in patients with solid tumors (including SS); nevertheless, no SS-specific analyses were conducted.^13^ Finally, three studies have shown no impact on OS. In a Cancer Genome Atlas-based study, OS in patients with SS was not affected by NY-ESO-1 expression; however, this analysis was only based on 10 patients with SS.^12^ In a study investigating the expression of NY-ESO-1 by IHC and real-time polymerase chain reaction in SS, Iura et al.^15^ reported a strong correlation between high NY-ESO-1 expression and the presence of necrosis and advanced clinical stage; nevertheless, the study was limited by a lack of survival data and the small number of SS samples (*n* = 11) used for gene expression profiling. Our findings align with the recent study from Gyurdieva et al., in which OS was not affected by NY-ESO-1 expression in 45 patients with SS.^16^ To our knowledge, the present study is one of the largest clinico-biological cohort studies specifically focusing on the prognostic value of NY-ESO-1 in patients with mSS, a rare subtype of STS.

HLA-A∗02 positivity had no prognostic effect on OS in the current study, consistent with a recent study in which patients with mSS underwent HLA typing to determine eligibility for a clinical trial of NY-ESO-1-specific engineered T cells, and reported that HLA-A∗02:01, HLA-A∗02:05, and HLA-A∗02:06 genotypes did not have a prognostic effect on OS in patients with mSS.^14^

Retrospective studies are generally associated with certain limitations, including possible bias from missing data in medical records, possible inconsistencies within and across physician assessments and radiological reports, underpowered estimates that may be unreliable due to the rarity of SS, and age of samples. In our study, both the primary and exploratory cohorts had very few missing IHC patient data; any data missing from the NetSARC database were obtained by contacting treatment centers and accessing individual patient electronic medical records. The possible bias occurring from the low sample size due to the rarity of the disease and available metastatic tissue samples was minimized with the inclusion of the exploratory cohort analysis. Moreover, based on a sample size of 100, it was estimated that the maximum HR yielding a significant difference in survival between positive and negative subgroups for NY-ESO-1 was 0.60. This threshold was surpassed in our study, ensuring the possible bias of underpowered estimates due to the rarity of SS was minimized.

Overall, our findings provide further clarity on the lack of prognostic impact of NY-ESO-1 expression in a broad population of patients with mSS and expected clinical outcomes in this population. The results of this study can inform trial design in this rare population, for novel treatments that target NY-ESO-1 with or without HLA restriction.

### Conclusion

To our knowledge, this is the first study to examine the prognostic impact of both NY-ESO-1 and HLA-A subtype in patients with mSS, and the first study in this population using both real-world data and archival tissue samples. This study provides much-needed information on outcomes in mSS, a distinct and rare subset of the broader STS population. NY-ESO-1 and HLA-A type, independently and together, show no significant impact on OS. Prognosis remains poor for this population, regardless of NY-ESO-1 expression and HLA-A subtype, and there is a need for new treatments that improve OS. NY-ESO-1 remains an important target in mSS, and this study can inform the selection of relevant populations of patients for clinical trials and trial design.

## Figures and Tables

**Supplementary Figure 2:**