# Supplementary material for: Investigating the prognostic impact of NY-ESO-1 expression and HLA subtypes in metastatic synovial sarcoma
Source: ESMO Open. 2024 Aug 16;9(8):103645. doi: 10.1016/j.esmoop.2024.103645 (PMC11381441; doi:10.1016/j.esmoop.2024.103645)
Supplement: Supplementary data [file mmc1.docx]

# Supplementary data

# Supplementary methods

## *Patient data protection*

Centre Léon Bérard (CLB) certified that patients included in this study did not oppose the re-use of their data, and this study followed all the processing steps with regard to General Data Protection Regulation (GDPR) and French regulations, including declaration of the data processing by the project coordinator, simplified risk analysis by the data protection officer, registration in the GDPR directory of the institution, establishment of patient information modalities, and declaration of the data processing on the French Data Transparency Portal for patients. The study has been validated by the CLB executive committee on December 3, 2019, under reference R201-004-012.

## *Tissue collection and assay validation*

Available formalin-fixed paraffin-embedded (FFPE) primary tumor samples were collected by the Biological Resources Center of CLB (“BioBanking Platform”). The biopathology department (sarcoma expert pathologist from the “Reseau de Référence en Pathologie des Sarcomes” [RRePS] network) of CLB reviewed each case to confirm the pathological diagnosis and assessed the quality and quantity of tumor samples. In cases of insufficient tumor sample available for both New York esophageal squamous cell carcinoma 1 (NY-ESO-1) and human leukocyte antigen (HLA) testing, NY-ESO-1 testing was prioritized.

Validation of the CLB NY-ESO-1 (E978 Sigma antibody) immunohistochemistry (IHC) assay was conducted to establish staining performance and intra- and inter-day precision assessments on a Leica Bond automated IHC staining platform. All acceptance criteria, as outlined in the assay validation plan, were met, demonstrating robust and reproducible detection of NY-ESO-1 protein in FFPE specimens.

In addition, a concordance assessment between the CLB NY-ESO-1 assay and the GSK Clinical Trial Assay (CTA) used in Study 208467 was performed on a cohort of 27 synovial sarcoma specimens (16 positive and 10 negative, one not evaluable) to determine positive percent agreement, negative percent agreement, and overall agreement point estimates, which were all above the acceptance threshold of ≥85%, thus demonstrating sufficient concordance to the CTA to enable the use of the CLB assay in the prognostic study.

## *Concordance assessment of HLA type between tumor tissue and blood*

Although NY-ESO-1 expression detection was assessed on tissue samples via IHC, HLA type expression was determined by RNA-seq in tumor tissue rather than blood. To ensure HLA typing in tissue using next-generation sequencing was concordant with HLA typing in blood, we conducted a separate exploratory study comparing the results of detection methods in blood and tissue on the RNA and DNA levels. All comparisons concluded that HLA detection in the tissue was highly concordant with detection in blood (40/41 samples; 97.6%) and is supportive of the methodology used for HLA determination in this study.

Supplemental Figure 1. OS according to NY-ESO-1 positivity by IHC in exploratory cohort.


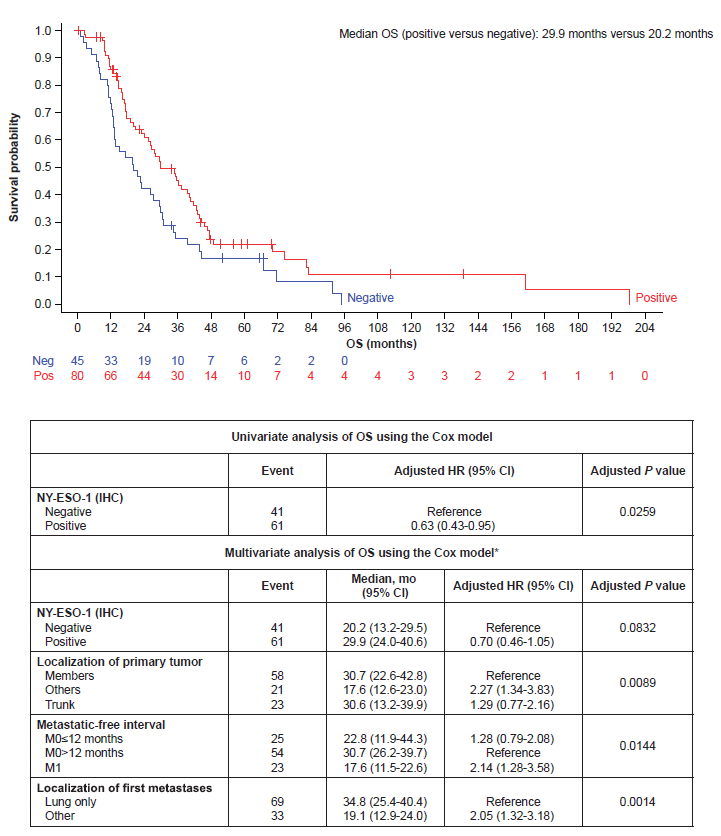


CI, confidence interval; HR, hazard ratio; IHC, immunohistochemistry; mo, months; NY-ESO-1, New York esophageal squamous cell carcinoma 1; OS, overall survival; SS, synovial sarcoma. ^a^Final model is estimated on 124 patients and 102 deaths due to missing values. All multivariate analysis *P* values were calculated by Wald Chi-Squared test.

Supplemental Figure 2. OS according to HLA-A*02 positivity in primary (A) and exploratory (B) patient populations (with unadjusted HR and *P* values).


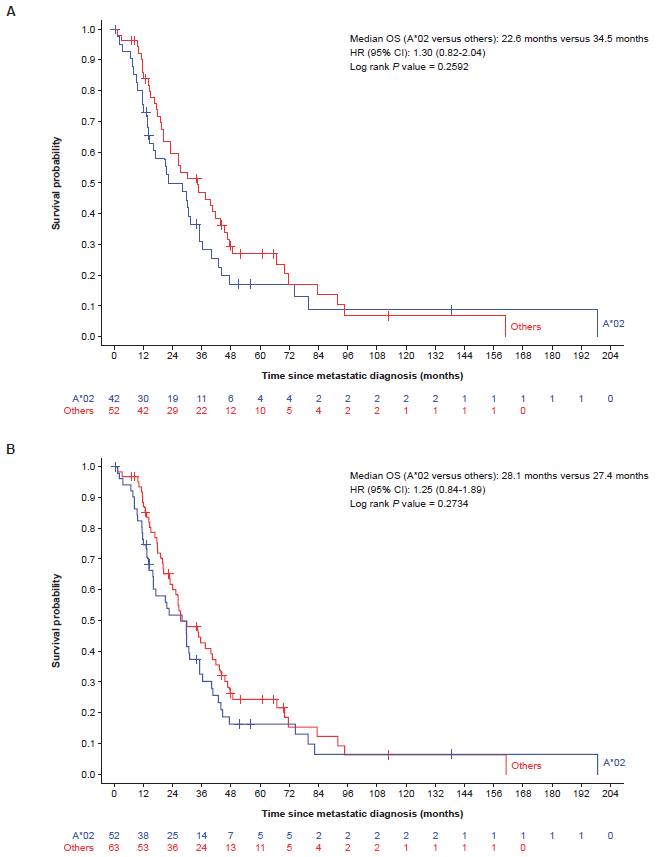


CI, confidence interval; HLA-A, human leukocyte antigen-A; HR, hazard ratio; NY-ESO-1, New York esophageal squamous cell carcinoma 1; OS, overall survival.
HLA-A*02 (HLA+) includes patients with HLA-A*02:01, HLA-A*02:05, or HLA-A*02:06 status.
Others includes patients who are negative for HLA-A subtype A*02, and either positive or negative for NY-ESO-1 expression.

Supplemental Table 1. Summary of NY-ESO-1 expression by IHC analysis and HLA-A status for the 14 patients with paired samples of primary and metastatic sample.

|  | | **NY-ESO-1 biomarker status (IHC) – 14 pairs** | | | | | **Treatment before sampling** | |
| --- | --- | --- | --- | --- | --- | --- | --- | --- |
|  |  | **Primary** | | **Met** | | **Biomarker status variation** |  | |
| **Patient ID** | **Metastasis localization** | **Biomarker status** | **H-score** | **Biomarker status** | **H-score** |  | **Primary** | **Met** |
| P099 | Lung | Neg | 30 | Pos | 150 | Increased in met | No | Systemic treatment doxorubicin + ifosfamide |
| P101 | Lung | Neg | 0 | Pos | 150 | Increased in met | No | Systemic treatment ifosfamide |
| P104 | Lung | Neg | 30 | Neg | 15 | Stable | No | Systemic treatment trabectedin |
| P111 | Lung | Pos | 250 | Pos | 170 | Stable | No | No |
| P122 | Lung | Pos | 300 | Pos | 300 | Stable | No | Systemic treatment doxorubicin + ifosfamide |
| P133 | Lung | Neg | 30 | Neg | 0 | Stable | No | Systemic treatment epirubicin + ifosfamide |
| P139 | Lung | Pos | 180 | Neg | 0 | Decreased in met | No | Systemic treatment doxorubicin + ifosfamide |
| P141 | Lung | Pos | 300 | Pos | 250 | Stable | No | Systemic treatment epirubicin + ifosfamide |
| P142 | Lung | Pos | 170 | Pos | 300 | Stable | No | Systemic treatment doxorubicin + ifosfamide |
| P145 | Lymph node | Pos | 240 | Pos | 235 | Stable | No | Systemic treatment doxorubicin + ifosfamide |
| P148 | Lung | Pos | 297 | Pos | 300 | Stable | No | No |
| P166 | Bone | Neg | 0 | Pos | 100 | Increased in met | No | Systemic treatment doxorubicin + ifosfamide |
| P171 | Other | Pos | 210 | Pos | 170 | Stable | No | Systemic treatment cisplatin + doxorubicin + ifosfamide |
| P178 | Lymph node | Neg | 30 | Neg | 80 | Stable | No | Systemic treatment cisplatin + doxorubicin + ifosfamide |

HLA-A, human leukocyte antigen-A; IHC, immunohistochemistry; Met, metastatic; Neg, negative; NY-ESO-1, New York esophageal squamous cell carcinoma 1; Pos, positive.

Supplemental Table 2. OS in patients with mSS receiving at least one line of systemic treatment.

|  | **NY-ESO-1+ versus NY-ESO-1−** | |  |  |
| --- | --- | --- | --- | --- |
| **Cohort** | **Number of events/*N*** | **Median OS (months)** | **HR (95% CI)** | ***P* value** |
| **Primary cohort** | | | | |
| NY-ESO-1 expression (unadjusted) | 43/57 versus 35/38 | 26.2 versus 17.0 | 0.57 (0.36-0.90) | 0.0137 |
| NY-ESO-1 expression (multivariate analysis) |  |  | 0.64 (0.39-1.04) | 0.0708 |
| NY-ESO-1 expression and HLA-A*02 (univariate analysis) | 16/19 versus 54/64^a^ | 33.1 versus 18.9^a^ | 0.65 (0.36-1.16) | 0.1447 |
| **Exploratory cohort** | | | | |
| NY-ESO-1 expression (unadjusted) | 56/71 versus 37/40 | 22.8 versus 16.0 | 0.57 (0.37-0.86) | 0.0076 |
| NY-ESO-1 expression (multivariate analysis) | 56/71 versus 37/40 |  | 0.52 (0.34-0.82) | 0.0041 |
| NY-ESO-1 expression and HLA-A*02 (univariate analysis) | 21/24 versus 64/76^a^ | 26.2 versus 20.5^a^ | 0.76 (0.45-1.26) | 0.2786 |

CI, confidence interval; HLA-A, human leukocyte antigen-A; HR, hazard ratio; IHC, immunohistochemistry; mSS, metastatic synovial sarcoma; NY-ESO-1, New York esophageal squamous cell carcinoma 1; OS, overall survival.
NY-ESO-1 expression is determined by IHC.
^a^Values show data for patients who were positive for NY-ESO-1 and had HLA-A subtype A*02 versus those who were negative for NY-ESO-1 and had other HLA-A subtypes.

Supplemental Table 3. OS in patients with mSS receiving at least two lines of systemic treatment.

|  | **NY-ESO-1+ versus NY-ESO-1−** | |  |  |
| --- | --- | --- | --- | --- |
| **Cohort** | **Number of events/*N*** | **Median OS (months)** | **HR (95% CI)** | ***P* value** |
| **Primary cohort** | | | | |
| NY-ESO-1 expression (unadjusted) | 36/38 versus 25/27 | 10.0 versus 9.3 | 0.90 (0.53-1.51) | 0.6796 |
| NY-ESO-1 expression (multivariate analysis) | 35/37 versus 23/25 |  | 0.97 (0.57-1.66) | 0.9117 |
| NY-ESO-1 expression and HLA-A*02 (univariate analysis) | 13/13 versus 42/46^a^ | 12.9 versus 10.2^a^ | 0.68 (0.35-1.33) | 0.2526 |
| **Exploratory cohort** | | | | |
| NY-ESO-1 expression (unadjusted) | 45/48 versus 28/29 | 10.0 versus 6.9 | 0.81 (0.50-1.31) | 0.3834 |
| NY-ESO-1 expression (multivariate analysis) | 44/47 versus 26/27 |  | 0.92 (0.56-1.51) | 0.7371 |
| NY-ESO-1 expression and HLA-A*02 (univariate analysis) | 16/16 versus 51/55^a^ | 11.9 versus 10.0^a^ | 0.72 (0.40-1.31) | 0.2762 |

CI, confidence interval; HLA-A, human leukocyte antigen-A; HR, hazard ratio; IHC, immunohistochemistry; mSS, metastatic synovial sarcoma; NY-ESO-1, New York esophageal squamous cell carcinoma 1; OS, overall survival.
NY-ESO-1 expression is determined by IHC.
^a^Values show data for patients who were positive for NY-ESO-1 and had HLA-A subtype A*02 versus those who were negative for NY-ESO-1 and had other HLA-A subtypes.
